# First person – Peter Szaraz

**DOI:** 10.1242/bio.046136

**Published:** 2019-07-15

## Abstract

First Person is a series of interviews with the first authors of a selection of papers published in Biology Open, helping early-career researchers promote themselves alongside their papers. Peter Szaraz is first author on ‘A solution to prevent secondary flow in adherent cell cultures’, published in BiO. Peter is a Research Associate in the lab of Clifford L. Librach at the University of Toronto, Canada, investigating *in vitro* techniques to test and preserve the regenerative potential of cell therapy candidates.


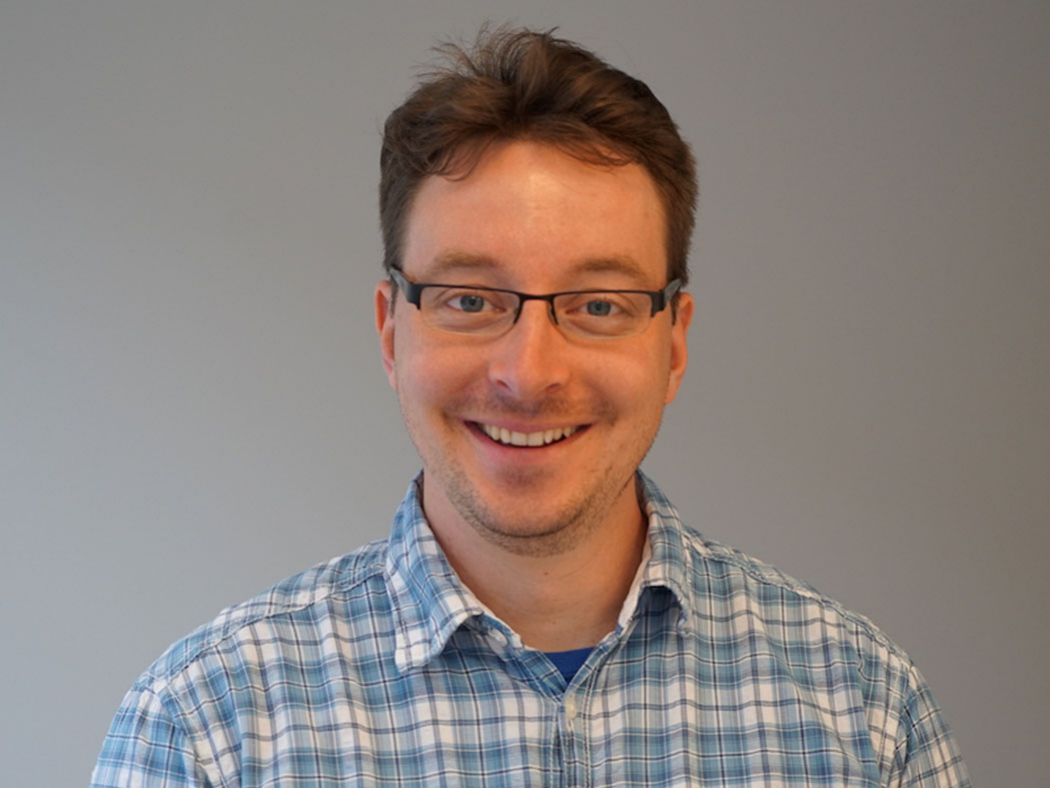


**Peter Szaraz**

**What is your scientific background and the general focus of your lab?**

My scientific background is cell biology, with special focus on cell fate decisions, lineage commitment and programmed cell death mechanisms. In particular, I am interested in testing and preserving the regenerative potential of stem cells and progenitor cells, and establishing bioreactor cultures, aiming for the development of cell therapy applications in the clinical setting. In our laboratory we mainly assess the regenerative potential of umbilical cord-derived angiogenic stem cells for various ischemia-related diseases.

**How would you explain the main findings of your paper to non-scientific family and friends?**

There is much more cell biology research happening worldwide than the actual medicinal applications realized from it. I found that the way we handle and grow cells in a dish has a big impact on the behavior of the cells and their usefulness as medicine. All around the world we still use so-called Petri dishes, which can cause big differences between results lab-to-lab because of how cells grow in them. With a simple modification of our cell culture practice, I could achieve better cell cultures and more replicable results that make the understanding and developing of regenerative cells faster and more reliable.

**What are the potential implications of these results for your field of research?**

More reliable cell cultures take us closer to high-scale production of regenerative cells, while preserving their medicinal potential. This can make the field of small-scale cell culture evaluations more consistent and accelerate the discovery and translation of new therapeutical cell types for clinical applications.

“More reliable cell cultures take us closer to high-scale production of regenerative cells, while preserving their medicinal potential.”

**What has surprised you the most while conducting your research?**

Beyond the impact of the new culture strategy on cell growth and behavior, the most remarkable feature was the fundamental simplicity of our experimental approach. The phenomenon that can cause so much challenge and hardship for many who culture cells for basic or translational research has been known for over a hundred years, yet no one changed the practices the way we did.

**What, in your opinion, are some of the greatest achievements in your field and how has this influenced your research?**

The development of bioreactor cultures brought much more control into cell culture practices and made it possible to generate enough cells in a homogenous way to even treat people with degenerative diseases. While it advanced the field greatly, it also generated a big gap between the quality small-scale generated cell cultures and large-scale cell production. We struggled with his issue ourselves, which drove our research to find a solution to close the ‘quality-gap’ and achieve better small-scale cultures that precede and successfully match bioreactor environment.

**What changes do you think could improve the professional lives of early-career scientists?**

Scientists in their early careers often struggle with the hectic nature of their job and lifestyle. The control over their career and making the right decisions requires a working knowledge of their field, not only scientifically, but of the opportunities to gain good training and to find their place in the academic or industrial job market. Many find that the postdoctoral stage is the bottleneck of our career, with low income and often low scientific credit to be gained. Better recognition of the ‘postdoc-society’, who make up the most vital functional layer of the scientific community could both improve the overall output of the research society and keep talented people from leaving their passion for other jobs where their investment translates more easily to better life quality.

**Figure:**
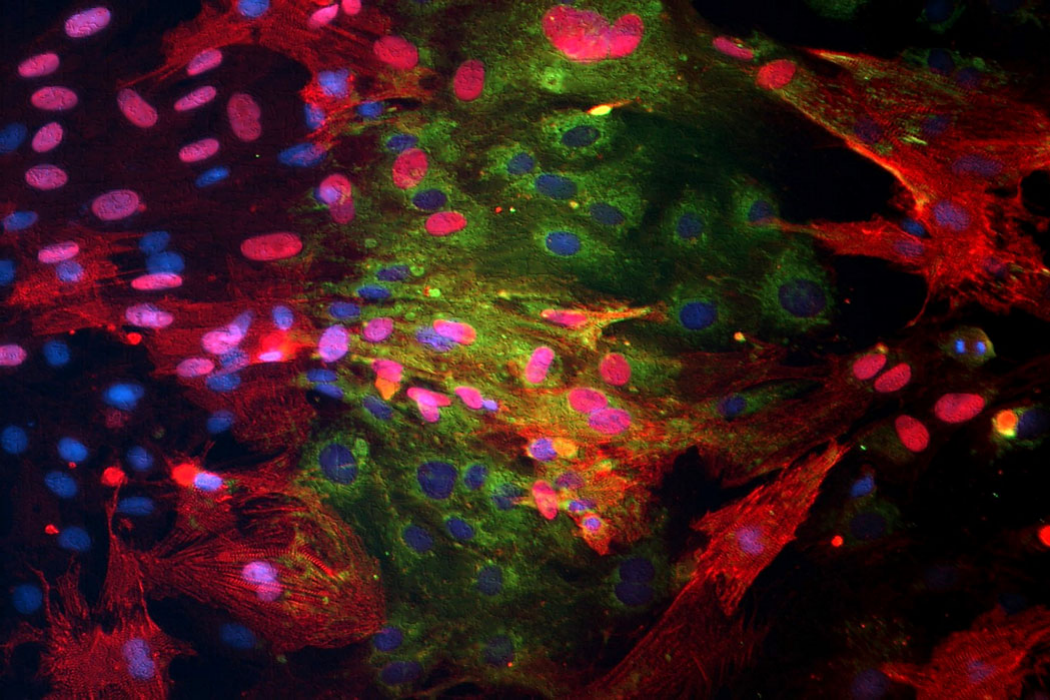
**Human umbilical cord stem cells cultured with primary rat heart cells – human cells assume the identity and phenotype of hosting tissue.**

**What's next for you?**

I am lucky to be involved in research that makes me feel that our work is ultimately resulting in the development of clinical applications. Our most recent collaboration with the Ottawa Hospital Research Institute brings that to a close reach. My dream is to have my own laboratory that focuses on the assessment of implantable regenerative cells and advances their testing and clinical translation.
